# Point-of-Care Ultrasound in the Primary Care Office for Early Detection of Squamous Cell Carcinoma of the Supraglottic Larynx: A Case Report

**DOI:** 10.7759/cureus.65682

**Published:** 2024-07-29

**Authors:** James Wilcox, Samuel L Kaefer

**Affiliations:** 1 Department of Family Medicine, Indiana University School of Medicine, Indianapolis, USA

**Keywords:** point-of-care ultrasound, early detection, squamous cell carcinoma, primary care, ultrasound

## Abstract

Point-of-care ultrasound (PoCUS) in the head and neck region is still in its early stages, despite decades of formal ultrasound use. A literature gap exists as recent studies primarily focus on general techniques, leaving room for exploration in ambulatory primary care, especially regarding neck masses. Our case demonstrates a 61-year-old female who presented for an annual wellness appointment, reporting a cough and alarming neck symptoms. A prompt investigation using PoCUS identified a suspicious neck mass. This mass was evaluated with bedside POCUS, and the mass was determined to be an abnormal lymph node with findings concerning malignancy. Subsequent diagnostic measures confirmed metastatic squamous cell carcinoma in the supraglottic larynx. This case underscores PoCUS's transformative potential in ambulatory primary care for neck mass evaluation, facilitating swift and thorough diagnostic processes. This successful outcome emphasizes PoCUS's promising role in routine clinical practice, urging future research for standardized evaluation protocols to enhance diagnostic efficiency.

## Introduction

Despite the use of formal ultrasound in the head and neck region for many decades, the specific use of point-of-care ultrasound (PoCUS) within this area of the body appears to still be in its early stages as evidenced by much of the recent literature still focusing on general technique and usage [1-3]. Bedside imaging with ultrasound evaluation, PoCUS, has been utilized for decades in the trauma setting. Recently, the application of bedside imaging with ultrasound expanded in use for clinicians outside of the trauma setting. Primary care clinicians are now using ultrasound for initial triage in several situations. However, the potential utility of PoCUS in the neck region can be easily ascertained through a quick review of the literature, including aiding in the diagnoses of peritonsillar abscess or Ewing sarcoma in the emergency department, evaluating thyroid in subspecialty ambulatory clinic, evaluating airway management in the intensive care unit, or pre-operative intubation evaluation [4-8]. Of note, PoCUS has been found to have high reliability in diagnosing neck masses in pediatric emergency rooms [9]. Despite the growth of PoCUS usage in the ambulatory primary care setting, head and neck scanning, with the exception of thyroid PoCUS, has not yet been widely reported. Additionally, there has been widespread study and characterization of malignant head and neck lymph nodes and laryngeal cancer in the form of formal ultrasounds within the field of Otolaryngology and Head and Neck Surgery; however, to the best of our knowledge, none of the existing literature focuses on the use of PoCUS [10-12]. Head and neck masses are difficult to evaluate in the primary care setting, as many tools for evaluation like laryngoscopes, PET-CT imaging, and ultrasound are not always readily available to the clinician. However, with the increased utilization of PoCUS in primary care, faster triage and sometimes diagnosis are available for trained clinicians.

Herein, we report a case in which evaluation of a neck mass during an annual wellness appointment led to a swift escalation of care and decreased the time to eventual treatment of malignant pathology. To our knowledge, this is the first case reporting the use of PoCUS in the ambulatory primary care setting to extensively examine multiple neck lymph node levels and ultimately aid in the diagnosis of laryngeal squamous cell carcinoma. 

## Case presentation

Our patient was contacted in person about using her case and story for this case report, and the patient consented to use her story for the education of the medical community.

Our patient was a 61-year-old female with a past medical history significant for 50+ tobacco pack year history, hypertension, anxiety, lupus, Sjogren’s syndrome, vitamin D deficiency, generalized unspecified chronic pain, and GERD who originally presented to our Federally Qualified Health Center (FQHC) clinic to discuss the desire to change blood pressure medications. Of note, this was the patient’s first in-person primary care appointment in over a year, and thus an annual exam was indicated. Upon conducting the patient interview, the patient described rather concerning newfound symptoms she had started having since her last primary care appointment. She noted daily hoarseness worse in the morning, which had been constant for a month, with a productive cough and associated dyspnea. Additionally, she described a new non-tender lump on her throat (palpable on physical exam) that was first noticeable three to four months prior to the clinic visit. In considering the patient’s new symptoms, the fact that this patient never had allergies requiring treatment, and an extensive smoking history, prompt and immediate investigation of the patient’s neck mass was deemed necessary.

**Figure 1:**
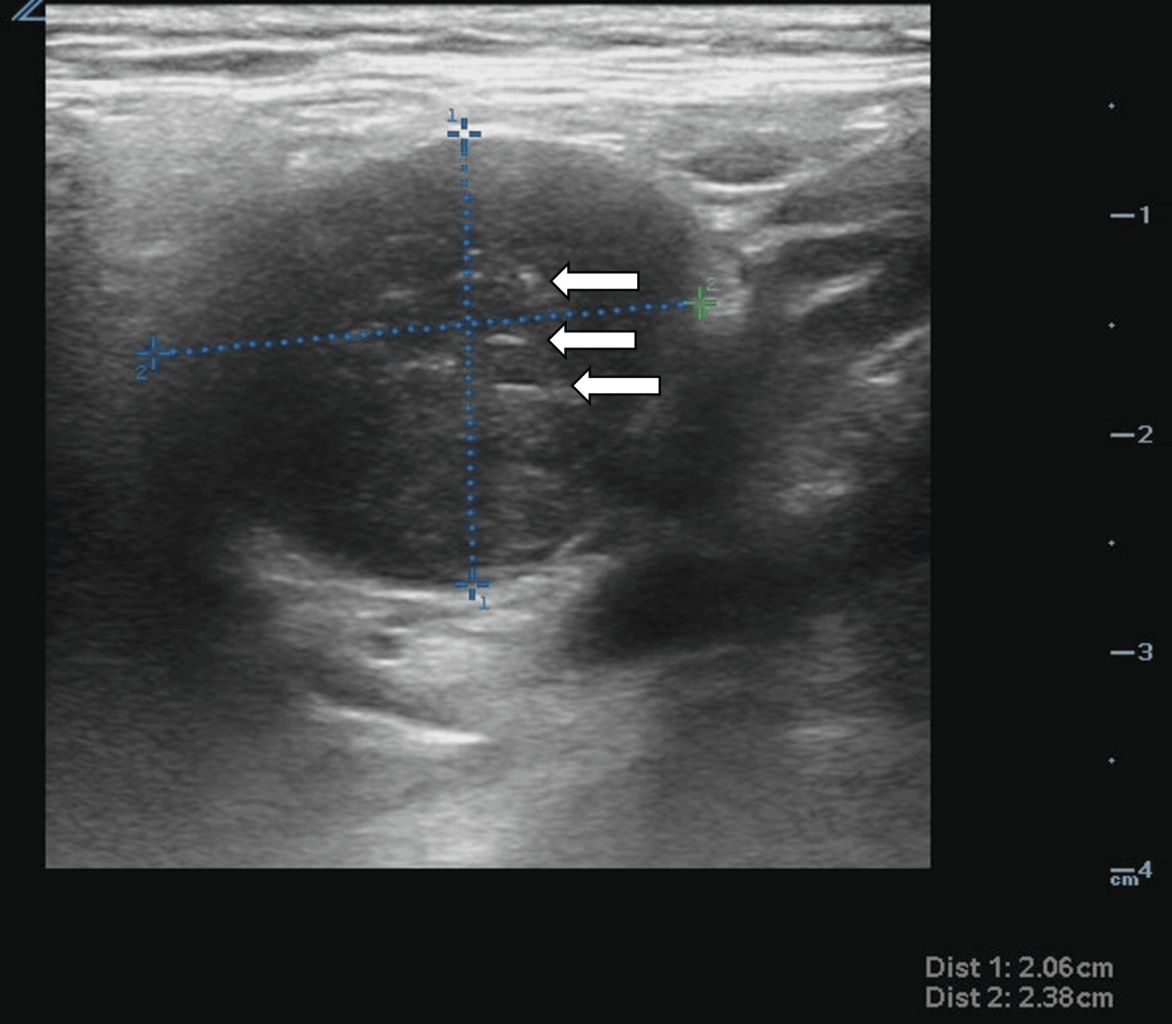
Enlarged cervical lymph node The ultrasound image shows an enlarged lymph node with a heterogenous appearance, obliteration of normal structure, increased blood flow on the Doppler, and a size of 2.1 x 2.4 cm. The mixed hyperechoic (white arrows) and isoechoic nature of the lymph node, added to the above other features like >2 cm size and abnormal structure, would mark this lymph node as "abnormal" in a typical PoCUS evaluation. An "abnormal" lymph node in point-of-care testing helps increase the clinician's suspicion of malignancy and guide which lymph nodes should be biopsied.

During the same initial primary care visit, PoCUS was utilized to further visualize the patient’s neck region. Following informed consent and a time-out, an in-office examination was performed utilizing a Zonare L10-5 Linear probe (Zonare Medical Systems, Inc, Mountain View, USA). The patient’s thyroid was imaged first. The left thyroid gland measured 1.3 cm in width by 1.1 cm in depth by 3.1 cm in length, with no obvious cysts or nodules appreciated. The isthmus measured 0.25 cm, with no obvious cysts or nodules appreciated. The right thyroid gland measured 1.7 cm in width by 1.8 cm in depth by 2.9 cm in length, and a small sub-centimeter mass measuring 0.4 cm by 0.6 cm was visualized. This mass was of mixed cystic and solid quality, isoechoic and anechoic, smooth, with no echogenic foci, and thus fell into TiRADS category two and required no follow-up. Additionally, it was quickly appreciable that the new lump the patient was describing did not stem from the thyroid. When the probe was moved to the left upper lateral portion of the neck (lymphatic level two), a distinct 2.1 cm by 1.9 cm mass resembling an enlarged lymph node (mixed isoechoic and hypoechoic circumferential mass with smooth walls) with internal blood flow on Doppler flow was appreciated, see Figure 1 and Video 1. This finding and the need for further workup were discussed with the patient, and an urgent referral was made for an otolaryngologist (ENT) consult as well as comprehensive neck ultrasound reading.

**Video 1:** Increased vascularity of the enlarged lymph node Video of the enlarged lymph node with multiple centers of Doppler flow signaling vascular neogenesis of the lymph node, denoting possible cancerous transformation.

**Figure 2:**
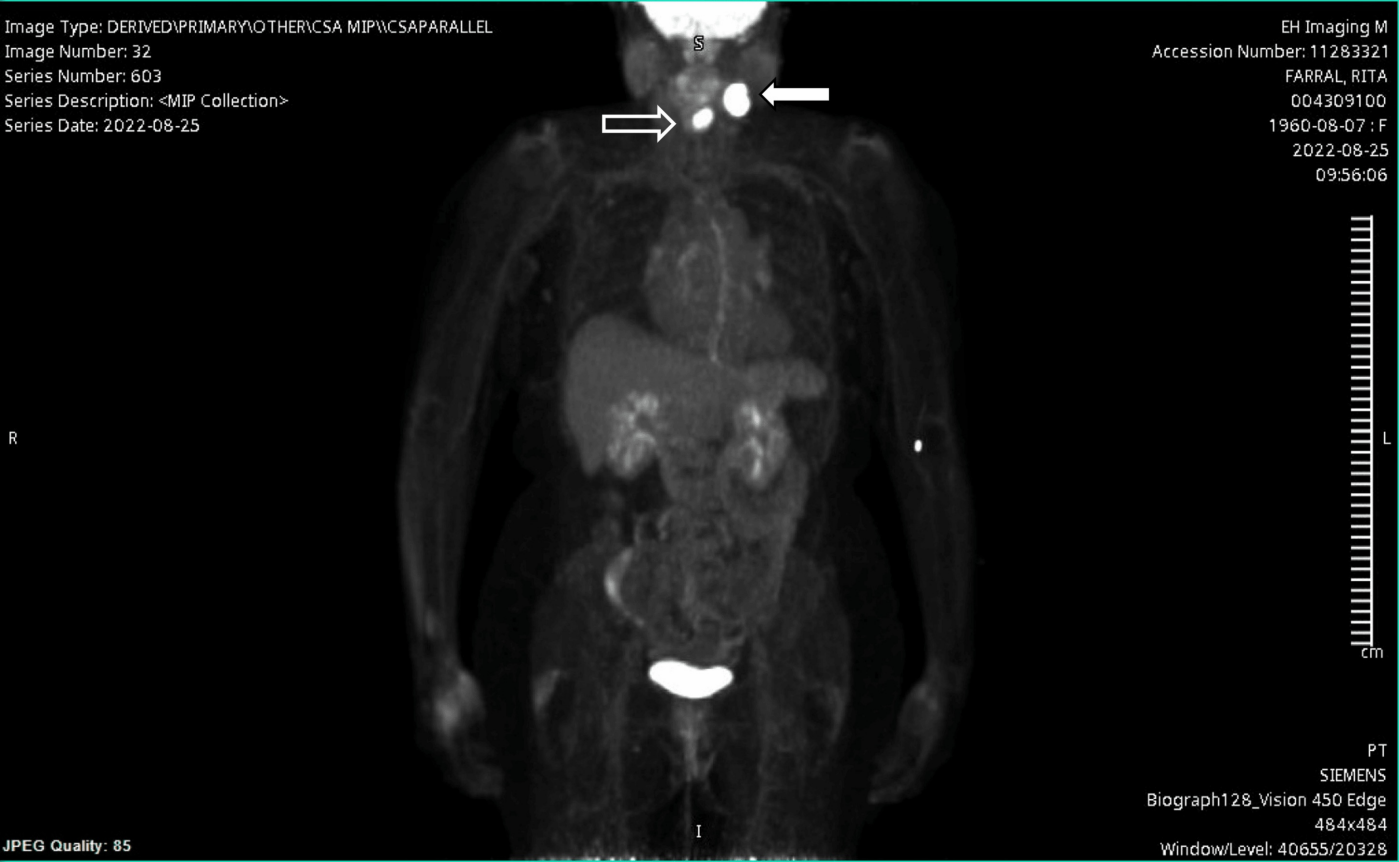
PET-CT of the laryngeal carcinoma and the neck mass This is the PET-CT of the laryngeal mass (open arrow) and the cervical neck mass (white arrow). These results confirm the laryngeal carcinoma location and demonstrate no other malignant spread other than the cervical lesion identified on PET-CT imaging. Identifying local spread helps determine treatment options for the treating surgery and oncology teams.

The patient was seen in the general ENT clinic roughly one month after the initial visit. At this point, dysphonia and lateral neck swelling symptoms had slightly worsened. During the ENT visit, a flexible video-stroboscopy was conducted. Although no exophytic lesions were seen on the vallecula, larynx, or hypopharynx, the left vocal fold was found to be completely immobile and supraglottic fullness was noted. Findings were discussed with the patient and within the setting of her smoking history, the decision to undergo further neck imaging (PET-CT) and fine needle aspiration (FNA) of the mass was agreed upon. The PET-CT is shown below in Figure 2.

**Figure 3:**
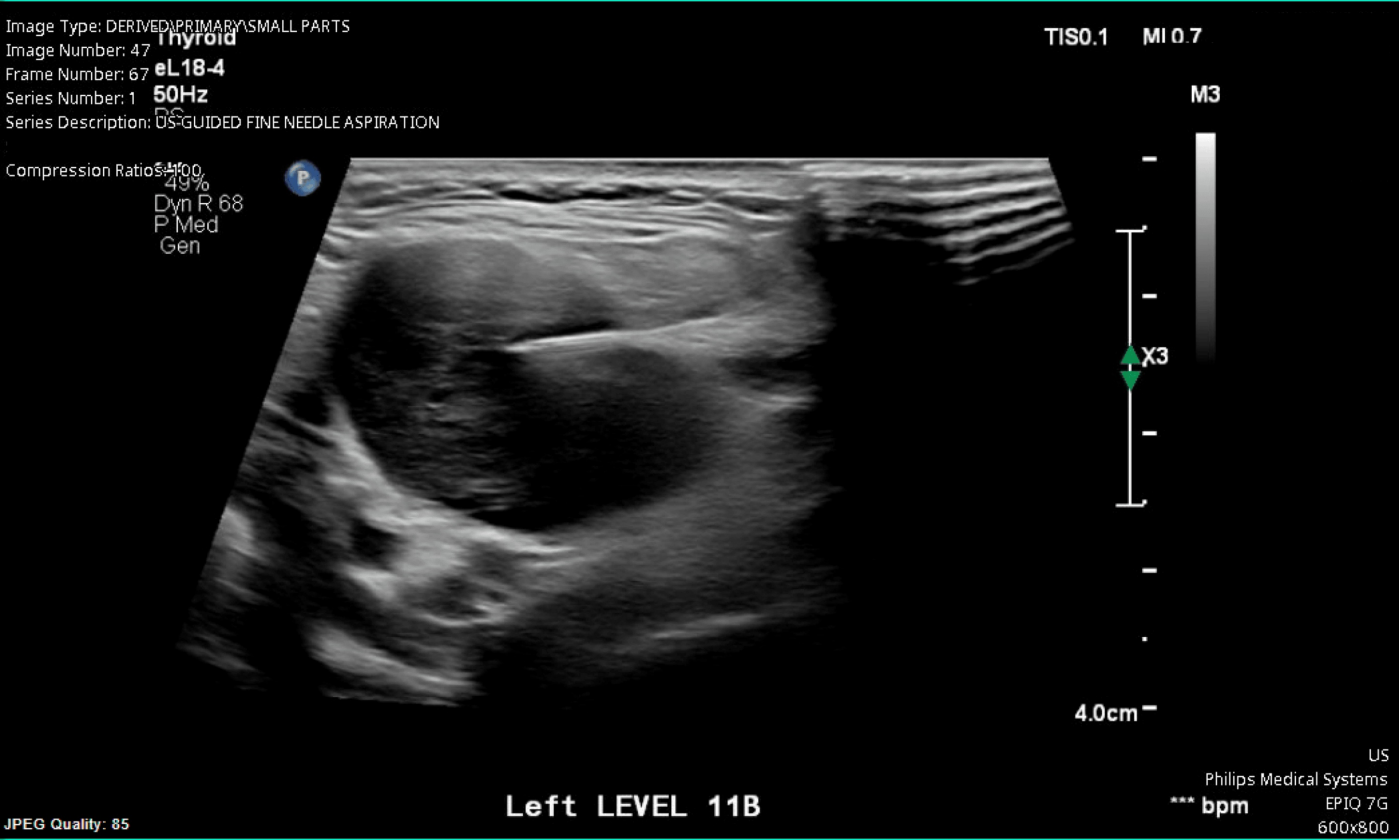
Ultrasound-guided FNA of the neck mass Ultrasound was used to guide the fine needle aspiration of the neck mass. The 18-gauge needle appears as a hyperechoic line, piercing into the center of the neck mass lesion. The needle is inserted into the lesion, and samples of the tissue are obtained percutaneously, and analyzed in a pathology laboratory for malignant or atypical cells. A biopsy is essential to determine if the malignant cells are differentiated or related to the current disease, or, in the unlikely case, if this is an unrelated primary malignancy of the lymph node. FNA: fine needle aspiration

Ultrasound-guided FNA of the patient’s neck mass, seen in Figure 3, demonstrated an abnormally appearing lymph node at the left level IIb, which was found to be metastatic squamous cell carcinoma p16+. PET-CT scan demonstrated a hypermetabolic mass involving the glottic and supraglottic larynx that extended into the paraglottic space involving lymph node levels IIa, IIb, and III. With the ultimate diagnosis of cT3cN2cM0 squamous cell carcinoma of the supraglottic larynx, the patient then met with various specialists within the head and neck cancer treatment field including head and neck surgery, oncology, radiation oncology to discuss potential treatment options (surgery versus chemo-radiation versus palliative treatment). The patient’s case was ultimately taken to the head and neck tumor board, and the recommendation for concurrent chemo-radiation therapy was made. The patient ultimately received treatment consisting of combined weekly cisplatin-radiotherapy for six weeks. Following treatment, she continued to be monitored by radiation oncology and head and neck at interval periods. One year following the completion of treatment continues to show no evidence of disease (in remission).

## Discussion

The integration of PoCUS into primary care settings has revealed significant potential for early detection and rapid management of critical pathologies such as squamous cell carcinoma (SCC) of the supraglottic larynx, as demonstrated in this case report. The early and precise detection of a neck mass in a 61-year-old patient led to timely diagnostic escalation and effective management, emphasizing the transformative potential of PoCUS in primary care [1-2].

Historically, ultrasound has been extensively used in the diagnosis of head and neck conditions, particularly by specialists in radiology and otolaryngology. It is especially effective in the differentiation of benign from malignant lymph nodes, where features like size, shape, and vascularity are key indicators of malignancy [10]. The novelty in our case lies in the use of ultrasound at the bedside, rather than comprehensive ultrasound in the radiology suite, in a primary care setting to initiate and expedite this diagnostic process. Additionally, since our case was seen in an FQHC and our patient had previously demonstrated a low desire for interaction with the healthcare community, this case highlights the ability to impress on patients the importance of getting further evaluation or treatment for a disorder and increase patient satisfaction with clinician care, as seen in other reports [13].

This case also aligns with recent findings that underscore the high reliability and utility of PoCUS for identifying abnormal neck masses in settings such as pediatric emergency rooms [9]. In the realm of SCC of the supraglottic larynx, a particularly aggressive form of cancer, the speed at which a diagnosis can be reached and treatment initiated is paramount to patient survival. PoCUS in our case facilitated a faster diagnostic timeline than typically achieved through traditional referral pathways to specialty care, which often involve prolonged waiting periods for imaging and consultations.

## Conclusions

In conclusion, this case report underscores the potential transformative role of PoCUS in the ambulatory primary care setting, particularly in the evaluation of neck masses. Through a comprehensive examination using PoCUS, we identified a suspicious neck mass in a patient with alarming symptoms, leading to a swift and thorough diagnostic workup, which the patient was motivated to complete. The use of PoCUS facilitated early detection and subsequent timely intervention, ultimately resulting in the diagnosis of laryngeal squamous cell carcinoma. To our knowledge, this is the first reported case demonstrating the valuable contribution of PoCUS in extensively examining multiple neck lymph node levels to aid in the diagnosis of head and neck malignancy in the primary care setting. The patient's successful outcome, with no evidence of disease one year post-treatment, highlights the impact of early detection and collaborative decision-making in the management of head and neck cancers. In future research, a standardized evaluation protocol could be developed to efficiently improve diagnostic outcomes.

This case serves as a noteworthy example of the expanding role of PoCUS in the primary care setting and emphasizes its potential to expedite the diagnostic process for critical conditions, ultimately improving patient outcomes. As PoCUS continues to evolve, its integration into routine clinical practice may prove instrumental in enhancing the efficiency of early disease detection, particularly in the realm of head and neck pathology.
